# SWIGH-SCORE: A translational light-weight approach in computational detection of rearranged immunoglobulin heavy chain to be used in monoclonal lymphoproliferative disorders

**DOI:** 10.1016/j.mex.2024.102741

**Published:** 2024-05-10

**Authors:** Marcus Høy Hansen, Markus Maagaard, Oriane Cédile, Charlotte Guldborg Nyvold

**Affiliations:** a Haematology-Pathology Research Laboratory, Research Unit of Haematology, Department of Hematology, and Research Unit of Pathology, Department of Pathology, University of Southern Denmark and Odense University Hospital, Odense, Denmark; b OPEN, Odense Patient data Explorative Network, Haematology-Pathology Research Laboratory, Odense University Hospital, Odense, Denmark

**Keywords:** IGH rearrangements, Computational tool clonotyping, Myeloma, Lymphoma, Leukemia, B-cell malignancy, Diagnostics, Minimal residual disease, Measurable residual disease, Nanopore sequencing, Next-generation sequencing, Swigh-score clonotyping of rearranged immunoglobulins

## Abstract

We present a lightweight tool for clonotyping and measurable residual disease (MRD) assessment in monoclonal lymphoproliferative disorders. It is a translational method that enables computational detection of rearranged immunoglobulin heavy chain gene sequences.

• The *swigh-score* clonotyping tool emphasizes parallelization and applicability across sequencing platforms.
• The algorithm is based on an adaptation of the Smith-Waterman algorithm for local alignment of reads generated by 2nd and 3rd generation of sequencers.

For method validation, we demonstrate the targeted sequences of immunoglobulin heavy chain genes from diagnostic bone marrow using serial dilutions of CD138+ plasma cells from a patient with multiple myeloma. Sequencing libraries from diagnostic samples were prepared for the three sequencing platforms, Ion S5 (Thermo Fisher Scientific), MiSeq (Illumina), and MinION (Oxford Nanopore), using the LymphoTrack assay. Basic quality filtering was performed, and a Smith-Waterman-based *swigh-score* algorithm was developed in shell and C for clonotyping and MRD assessment using FASTQ data files. Performance is demonstrated across the three different sequencing platforms.

## Graphical abstract

**Figure:**
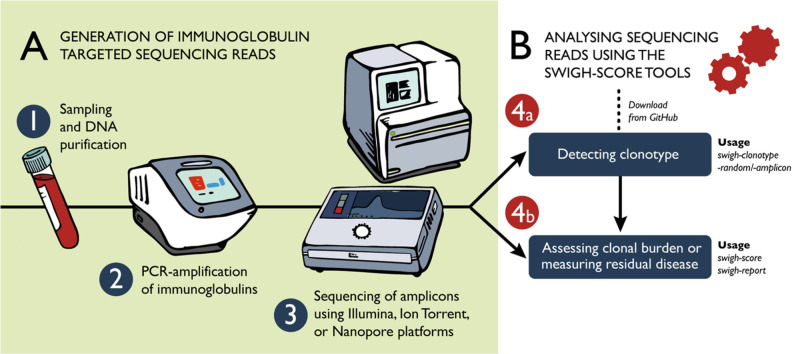

Specifications table

**Table:** 

Subject area:	Bioinformatics
More specific subject area:	Research of diagnostic methods in the detection of cancer of the blood, lymph nodes, and bone marrow
Name of your method:	Swigh-score clonotyping of rearranged immunoglobulins
Name and reference of original method:	Name of original method: Smith-Waterman algorithm **Reference of original method:** Smith TF, Waterman MS. Identification of common molecular subsequences. J Mol Biol. 1981 Mar 25;147(1):195–7. doi: 10.1016/0022–2836(81)90,087–5. PMID: 7,265,238.
Resource availability:	Scripts, C code, and demonstration video are available for download at the GitHub repository: **Link:**http://github.com/marcus-hoy-hansen/swigh-score Data used in this paper is found at figshare **Link**: http://dx.doi.org/10.6084/m9.figshare.24032322 *All steps of this paper can be reproduced using the downloadable scripts and data. Supporting information on is also found in referenced papers, for which the approach was first used* [1] *or patient clonotype thoroughly characterized* [2].

## Method details

In brief, this method represents a translational approach for identifying immunoglobulin heavy chain rearrangement to track residual disease in monoclonal B-cell lymphoproliferative disorders. It employs a powerful, though straightforward, parallelization strategy of local alignment, which can be directly implemented to screen patients with malignant clonal proliferation. The *swigh-score* tool refines and formalizes a method for clonotyping noisy and heterogeneous sequences, which was validated in characterizing eight multiple myeloma patient cases [1] and is adaptable to all common sequencing platforms. Given that these samples have been extensively characterized [2], we present data from a single patient sample to demonstrate multi-platform capabilities reproduced from nanopore sequencing, Ion Torrent, and Illumina-based sequencing. We refer to these papers for detailed documentation of clonotypes, residual disease detection validation, and reproducibility based on the LymphoTrack assay (Invivoscribe, San Diego, CA) using next-generation sequencing [2] and third-generation nanopore sequencing [1].

CD138-positive cells had been isolated from a bone marrow diagnostic sample using CD138 microbeads (StraightFrom Whole Blood CD138 microbeads, Miltenyi Biotec, Bergisch Gladbach, Germany) on an AutoMACS (Miltenyi Biotec). To assess the low-level detection, DNA was diluted into leukocyte DNA (10^−4^–10^−5^ dilution), consisting of a pool of DNA extracted from 13 control donor samples. The low-level assessment of replicates was only performed using Ion S5, as extensively detailed in the original paper [2].

Computations and tests were performed on the UCloud interactive HPC system, the eScience Center at the University of Southern Denmark, and a local 24-logical core workstation. All tools necessary for the analyses were developed as bash scripts or C code and run without any additional requirements than already available on the platform (Ubuntu 18–22 LTS or other Linux distribution), i.e., *gcc* (GNU Compiler Collection) compiler, and *awk*.

Immunoglobulin heavy chain Variable (V) and Joining (J) gene germ-line reference sequences were downloaded and implemented from the IMGT/V-Quest online resource (IMGT/GENE-DB reference sequences, www.imgt.org, *accessed April 20, 2023*) [[3], [4], [5]]. Clonotype predictions were tested against IMGT/V-Quest and IgBLAST [6].

## Quality filtering of sequencing reads

Prior to *IGH* clonotype detection or sequence similarity scoring, the raw sequencing files (FASTQ) underwent quality filtering. The default mean Phred-scaled quality score threshold was set to 20, i.e., a base-calling error rate threshold of 1 %. Also, only reads of at least 200 bp length were used for testing **(List. 1A)**.



**Listing 1A)**

An alternative to the default naive averaging of read quality scores (default run mode) is the mean base-calling error probabilities using the antilog function of the Phred score (run mode 2, **List 1B**).



**Listing 1B)**

### Smith-Waterman scoring

The implemented Smith-Waterman (SW) algorithm consists of a few simple steps. First, the scoring matrix with a dimension of *len1* × *len2*, representing the length of the two DNA strings (*str1, str2*), was populated with a binary comparison between nucleotides (**List. 2A**).



**Listing 2A)**

Next, looping through the rows and columns to find the most optimal local alignment by scoring substitution (**List. 2B**) was performed. Of note, the highest score in the matrix represents the starting points for backtracking (**List. 2C**), and it is necessary for calculating the similarity score adjusted to the length of the alignment (**List. 2D**). Only one tracing attempt was performed here, while multiple alignments may exist.



**Listing 2B)**



**Listing 2C)**



**Listing 2D)**

The presented algorithm was used for two separate purposes: **(1)** Clonotyping by suggesting the *IGH V*/*J* gene usage and **(2)** to find the number of sequences matching an identified clone at a specified threshold. In the former, the SW algorithm was put directly into action by investigating a single provided DNA sequencing using the *swigh-clonotype-sequence* bash script (**List. 3**).



**Listing 3)**

Clonotyping a single sequence is of limited use. Thus, the tools implement two other strategies for *IGH* clonotyping of diagnostic samples, i.e., *swigh-clonotype-amplicon* and *swigh-clonotype-random. Swigh-clonotype-amplicon* utilizes the fact that identical reads are frequent in amplicon-based sequencing (**List. 4A**).



**Listing 4A)**

As such, a clonal expansion of the immunoglobulins is rapidly identified by sorting and counting the unique reads (*sort* and *uniq* -c commands, **List. 4B**).



**Listing 4B)**

This amplicon approach is not optimal for the more diverse sequencing library generated by the Nanopore platform. Instead, *swigh-score-random* relies on stochastically subsampling the sequencing reads (*n* = 1000) with the subsequent prediction of V/J usage for each sequence (**List. 5**). This approach results in nearly half a million local alignments and is thus split into multiple processes akin to the *swigh-score* command outlined next, while not predicting V/J gene based on a similarity threshold. Also, it provides an alternative estimate of the clonal burden to the *swigh-score* command.



**Listing 5)**

### Shell wrapper of the Smith-Waterman scoring algorithm

The *swigh-score* script implemented parallel processing to estimate the clonal burden among rearranged cells in the diagnostic samples or to detect measurable residual disease (MRD) using an already identified clonotype sequence.



**Listing 6A)**

In brief, the script loads the quality-assessed reads, counts, and collapses the sequences **(List. 6B)**. Subsequently, the reads are split to perform pseudo-parallel multiprocessing, where the number of processes reflects vCPUs or logical cores available (n=vCPUs-1) on the machine for optimal performance **(List. 6C)**. That is, a FASTQ-file with millions of reads will undergo the same number of local alignment scoring divided into n processes (SWscoresequences.sh) using *n* file chunks.



**Listing 6B)**



**Listing 6C)**

Finally, the similarity scoring results were concatenated to a single output while preserving the number of unique read counts for readability (**List. 6D**).



**Listing 6D)**

The results were summarized using the *swigh-report* command for the generated *swigh-score* output file in a mixed tabulated format containing similarity distributions (**List. 7**).



**Listing 7)**

## Method validation

**Fig. 1:**
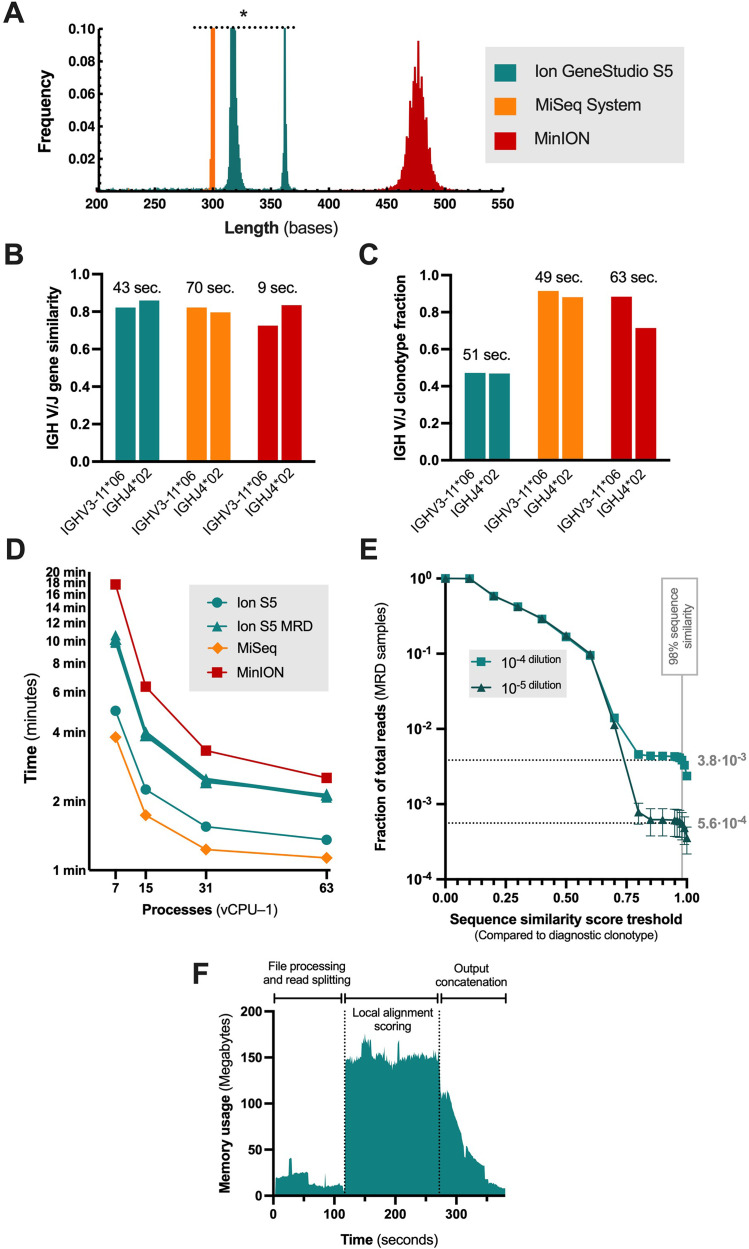
Analytical Results and Performance Metrics. Comparison of sequencing read lengths for the diagnostic sample obtained from different platforms: MiSeq, Ion S5, and MinION **(A)**. This frequency plot has deliberately been truncated for comparison (*). Clonotyping results using single-process *swigh-clonotype-amplicon*, showcasing sequence matches to *IGHV3–11×*06 and *IGHJ4×*02 in the diagnostic sample **(B)**. Clonal fraction estimation for different platforms using multiprocess *swigh-clonotype-random*, highlighting lower clonal fraction of Ion S5 relative to MiSeq and MinION **(C)**. Clonal fraction determination with the multiprocess Smith-Waterman algorithm (*swigh-score*) at 98 % sequence similarity threshold, revealing wall-time differences between Ion S5 and MiSeq due to higher sequence heterogeneity for Ion S5 in both diagnostic sample and the five samples used to demonstrate measurable residual disease detection (MRD, **D**). Pseudo-MRD assessment from serially diluted samples matched the expected 10-fold difference between the 10^−4^ and 10^−5^. Referring to these level, the clonal fraction corresponded to 38·10^−4^ and 56·10^−5^ at the 98 % similarity threshold **(E)**. Efficient memory usage was demonstrated for the *swigh-score* Smith-Waterman alignment analysis **(F)**.

Method validation was based on targeted sequencing of rearranged immunoglobulin heavy chain genes. For this, we applied a three-pronged detection setup from CD138+ enriched plasma cells purified from the diagnostic bone marrow of a patient with multiple myeloma, as profiled previously [2]. One baseline specimen was run on the three different platforms (0.2–1.6 million reads), and serial dilutions of the diagnostic sample (10^−4^–10^−5^-fold dilutions) were implemented for *pseudo-MRD* assessment (1 and 5 runs, respectively, obtaining 1.8–2.6 million reads using Ion S5). Quality filtering of the FASTQ files using the provided lightweight C code (*quality-filter*) processed 1 million reads in 7–8 s (*sec*), retaining reads of more than 200 bp length and mean Phred quality score threshold of *Q* ≥ 20. This threshold was selected to reflect a theoretical base-calling error rate of 1 % to differentiate between the homologous heavy-chain genes across the platforms (P_error_ ≤ 10^−^*^Q^*^/10^). The median MiSeq read length of the diagnostic sample was 301 bases (bp) versus 317 bp for Ion S5 and 476 bp for MinION (Fig. 1A). Although a fraction of reads may contain multiple base-calling errors, these are not considered detrimental when the overall clonotype assessment is based on a large population of reads and base-calling or PCR errors are assumed to be stochastic events.

Deep sequencing was performed on the Ion GeneStudio S5 System (Thermo Fisher Scientific, Waltham, MA, USA), MiSeq System (Illumina, San Diego, CA, USA), and MinION Mk1C (FLO-MIN106D flow cell, Oxford Nanopore Technologies, Oxford, UK) (see *Data availability*). Amplicon generation was based on the LymphoTrack *IGH* FR1 Assay for all sequencing modalities. Nanopore library preparation was performed using the Ligation Sequencing Kit (SQK-LSK110) on DNA derived from pooled Illumina MiSeq LymphoTrack amplicon DNA library. Thus, the patient-specific data was extracted by demultiplexing based on containing TruSeq Universal Adapter (Illumina), amplicon, and corresponding index adapter (i7, Illumina).

De–novo clonotyping of the diagnostic sample (**List. 6A**), using the presented SW implementations (*swigh-clonotype-amplicon* and *swigh-clonotype-random*), identified the monoclonal expansion harboring Variable gene *IGHV3–11×*06 and Joining gene *IGHJ4×*02 for all three sequencing platforms, in agreement with the clonotype provided by IgBlast and IGMT/V-Quest. This V/J gene usage also agreed with this patient's previous profile of CD138+ plasma cells (patient 1 [2]). Clonotyping using single-process *swigh-clonotype-amplicon* showed 73.5–83.2 % sequence match of the clone to *IGHV3–11×*06 and 80.6–87 % to *IGHJ4×*02 in 9–70 s runtime relative (Fig. 1B), which was relative to the number of reads.

The tool *swigh-clonotype-random* randomly assesses 1000 reads by default and assigned the correct clonotype in 49–63 s (63 processes). The approximate clonal fraction was markedly lower than expected for Ion S5, averaging 0.48 versus 0.86 collectively for MiSeq and MinION (Fig. 1C). Although this tool was constructed to define the most common *V*/*J* usage in a random subsample of reads, not directly assessing clonal burden, it may be practical for this purpose for more error-prone base-calling methods. Here, the *clonal burden* is defined as the relative fraction of clonally rearranged sequences to all detected sequences from IGH rearrangement. However, when a spike-in control sequence is implemented, as described below for the low-level detection, it becomes possible to provide an absolute estimate of clonal cells.

The defined clonotype sequence at 98 % sequence-similarity using the multiprocessing SW algorithm (*swigh-score*) amounted to 0.44 clonal fraction for Ion S5 and 0.85 for MiSeq (*swigh-report*) for the diagnostic sample with a run time of roughly 1–3 min per million analyzed reads (63 processes, Fig. 1D). Thus, these results, using all sequencing reads, agreed with *swigh-clonotype-random*.

The seeming low clonal burden for Ion S5 arose from exceeding sequence heterogeneity. Thus, filtering out highly dissimilar reads for both platforms (<25 % similarity) provided compatible results (0.90 versus 0.87). Optimally, the selected threshold for Nanopore sequencing reads must reflect the higher error rate [[7], [8], [9]], previously determined to be roughly 10 % with the same flow cell (FLO-MIN106D) type and library preparation kit [10], although in a whole-genome sequencing setup. Hence, the clonal fraction using 90 % down to 85 % sequence similarity to the clonotype was 0.77–0.97.

The samples prepared for pseudo-MRD assessment, i.e., factor 10^4^ (1 sample) and 10^5^ dilutions (5 replicates), showed a clonal fraction of 38·10^−4^ and 56·10^−5^ at the corresponding level (Fig. 1E, *swigh-score* and *swigh-report*, 98 % similarity threshold, 82.9 % *IGHV3–11×*06 match, 87 % *IGHJ4×*02 match). An increased processing time was observed for the MRD sample compared to diagnostic samples sequenced on Ion S5 and MiSeq (Fig. 1D) due to higher sequence heterogeneity, i.e., polyclonality. A spike-in control sequence, equivalent to 100 cells, was used to assess the clonal burden as an absolute measure. The estimated number of malignant cells was found to be 6.4 per 100,000 leukocytes for the 10^−4^ dilution and 50.9 per 100,000 for 10^−5^, concordant with the previous observations using the LymphoTrack assay and software for Ion S5 sequencing setup [2].

The memory consumption was less than 200 megabytes at any given moment (Fig. 1F), whereas the read/write operations (I/O) were intensive (data not shown). Because of this I/O bottleneck, the *swigh-score* wrapper of the SW implementation was tested on a local 24 logical-core workstation (Intel i9–9920X) using a RAM disk, i.e., a virtual hard drive created in the computer memory (*tmpfs* file system). The resulting processing time of the pseudo-MRD sequencing replicate containing the highest number of reads (2.6 million) was 324.8 s versus 321.1 s using the HPC 64 vCPU.

## Additional information

In this paper, we have presented a bioinformatics method and tool to test the capability of clonotyping and MRD detection using rearranged immunoglobulin heavy-chain genes in B-cell malignancies. This clonal profiling was validated by investigating CD138+ bone marrow cells from a patient diagnosed with multiple myeloma. The *swigh-score* tool provided here gives a basic, translational framework for clonotyping and MRD analysis across the different sequencing technologies platforms, MiSeq, Ion GeneStudio S5, and MinION. The tool is suitable for *IGH*-targeted sequencing only.

The main objective was to create a simple and parallelizable tool that can be used for screening patients with malignant clonal lymphoproliferation or complementing other software tools like the LymphoTrack assay. Although not as advanced as ARResT/Interrogate [11], developed by the competent and thorough EuroClonality-NGS working group, it is a powerful and straightforward approach that can be easily implemented and modified for research purposes. We aim to simplify the analytical process by reducing clonotyping and MRD assessment complexities using FASTQ data files generated from IGH-targeted DNA sequencing. The memory consumption of the tool is low but requires intensive I/O operations (read/write). However, loading sequencing data into memory (RAM disk) significantly improves the performance of the SW alignment using a local workstation. In either case, the tool can detect a clonal sequence among millions of reads in minutes with similarity scoring using SW local alignment.

Detection of MRD in patients with hematological malignancies has become an important surrogate marker for treatment efficacy [[12], [13], [14], [15], [16]]. The high specificity and precision of sequencing clonal immunoglobulin rearrangements within lymphoproliferative neoplasia offers a key advantage over alternative targets, such as the mutational burden of driver genes or transcriptional activity. The technique, i.e., clonotyping, serves a diagnostic purpose and holds prognostic value. Specifically, the assessment of unique clonal sequences can predict the likelihood of disease recurrence for lymphocytic neoplasia, such as acute lymphoblastic leukemia. Consequently, measuring MRD in patients with these forms of leukemia has become standard clinical practice **[**17**,**^18^**].**

Traditionally, MRD measurements have relied on PCR-based analysis or flow cytometry, each with strengths and limitations [12,19]. However, recent efforts have focused on harnessing next-generation sequencing (NGS) for MRD analysis, promising higher sensitivity and novel applications. This concept has been illustrated by Medina et al. [20], where NGS was used to profile high-frequency types of *IGH* genes in multiple myeloma. The EuroClonality-NGS Working Group likewise has performed technical feasibility and validation studies [21,22], proving the utility in profiling molecular targets for MRD measurements and demonstrating performance comparable with other MRD techniques and pointed to utilities such as the ability to discover clones more efficiently in a polyclonal background and to examine both clonal B-cell populations and immunoglobulin gene repertoires of individual patients more accurately.

Using NGS technology for MRD measurement is challenging. Stochastic variables influence the detection of MRD, and as such, the high sensitivities promised by NGS methods require ample testing material and proper, well-proportioned testing procedures tailored to the specific sensitivities. As described previously, comprehensive standardization and transparency are necessary to utilize NGS to quantify MRD measurements properly [2,23]. Furthermore, to be integrable into clinical contexts, clonotypes must first be identified, and amplification or sequencing error rates must not hinder the interpretation of the measurement [12].

The sequencing results must be interpreted to convey the necessary information to utilize NGS to its fullest potential toward MRD measurements. To accommodate this need, we developed a tool using the algorithm proposed by Smith and Waterman to identify the most homologous “*subsequences among sets of long sequences”* [24]. By employing process-level parallelization as suggested by others [25], we created an educational case-based framework to aid in using NGS and 3rd-generation, also known as long-read sequencing, for clonotyping and MRD interpretation.

MRD has evolved into a pivotal tool for evaluating treatment efficacy and predicting disease relapse in hematological malignancies. While MRD measurement traditionally relies on PCR-based analysis or flow cytometry, recent efforts have turned to NGS for its high sensitivity and specificity. Thus, further development and optimization of NGS-based MRD detection tools hold promise for improving disease management and treatment outcomes [12]. However, the application of NGS to MRD detection is challenging. Stochastic variables can affect MRD detection, necessitating standardized testing procedures [2,23].

The primary purpose of the developed *swigh-score* clonotyping tool is to facilitate the categorization and interpretation of FASTQ, the prevalent data format for presenting raw output from most DNA sequencing workflows. It offers commands that enable users to track the burden and development of a single clonotype, which can detect residual clonal B cells in various tissues, e.g., stem-cell products for autologous stem cell transplant [26] or blood plasma by sequencing cell-free tumor DNA [12,[27], [28], [29]]. Still, the utility and predictive value of MRD assessment varies among different diagnoses and cancer types, e.g., due to disease kinetics and available biomarkers, which can impact its clinical applicability [14].

When considering the sensitivity and specificity of MRD detection, it becomes evident that achieving high-resolution MRD detection places substantial demands on both sample quality and the analysis itself. All sequencing platforms have inherent error rates, and excessive emphasis on specific thresholds without further validation compromises the accuracy of the assays and clinical interpretations.

A low error rate is crucial when targeting low MRD levels, such as 10^−4^–10^−6^. While there have been attempts to use nanopore technology for MRD detection, such as the study by Sampathi et al. [30], small sample sizes limit these efforts and lack comparisons with more accurate sequencing platforms. However, the technology seems to be continuously refined, such as the currently available R10 flow cells and chemistry, suggesting that the sequencers might be an option for MRD assessment in the future.

In summary, the developed tool attempts to offer flexibility and provide information on clonotype similarity and distribution in FASTQ files. Its accuracy is intrinsically linked to the quality of underlying laboratory work, biological samples, and sequencing platforms. Post-analysis tools, like SW algorithm-based tools, are essential to harness NGS's full potential for clonotyping and MRD measurements. This tool aids in identifying homologous subsequences among long sequences and offers flexibility across different sequencing technologies.

## CRediT authorship contribution statement

**Marcus Høy Hansen:** Conceptualization, Methodology, Software, Validation, Formal analysis, Investigation, Resources, Data curation, Writing – original draft, Writing – review & editing, Visualization, Supervision, Project administration, Funding acquisition. **Markus Maagaard:** Methodology, Validation, Formal analysis, Investigation, Data curation, Writing – original draft, Writing – review & editing. **Oriane Cédile:** Formal analysis, Resources, Data curation, Writing – review & editing. **Charlotte Guldborg Nyvold:** Resources, Writing – review & editing, Supervision, Project administration, Funding acquisition.

## Declaration of competing interest

The authors declare that they have no known competing financial interests or personal relationships that could have appeared to influence the work reported in this paper.

## Data Availability

All data and code is linked directly in the manuskript (GitHub and figshare)

## Appendix Supplementary materials

Image, video 1
